# What Is the Effect on Kinesio Taping on Pain and Gait in Patients With Patellofemoral Pain Syndrome?

**DOI:** 10.7759/cureus.8982

**Published:** 2020-07-03

**Authors:** Alec S Kellish, Patrick Kellish, Abraham Hakim, Sandra Miskiel, Alisina Shahi, Allison Kellish

**Affiliations:** 1 Orthopaedics, Cooper Medical School of Rowan University, Camden, USA; 2 Psychiatry, Cooper Medical School of Rowan University, Camden, USA; 3 Physical Therapy, Professional Physical Therapy, Westfield, USA; 4 Internal Medicine, Cooper Medical School of Rowan University, Camden, USA; 5 Orthopaedics, Cooper University Hospital, Camden, USA; 6 Orthopaedics, School of Physical Therapy, Kean University, Elizabeth, USA

**Keywords:** gaitrite, patellofemoral pain syndrome, knee pain, physical therapy, gait, kinesiotaping, kinesio taping, gait analysis

## Abstract

Introduction

Patellofemoral pain syndrome (PFPS) is one of the leading causes of anterior knee pain treated by orthopedists and physical therapists. This syndrome predominantly affects young, active individuals, and remains a challenging syndrome to manage due to the lack of quantitative diagnostic criteria to monitor during treatment. The etiology of this syndrome is believed to be multifactorial, with the gait and movement patterns of a patient potentially contributing to pain due to increased stress on the knee. In this study, we investigated the gait of participants with PFPS using the GaitRite system (CIR Systems Inc., Clifton, NJ) before and after the application of Kinesio Tape in order to assess the impact of Kinesio Tape on cadence, stance time, and pain.

Methods

A convenience sample of 10 participants were recruited for this study, with five participants without PFPS serving as controls, and five with PFPS in the Kinesio Tape group. Participants in the Kinesio Tape groups served as their own internal control, ambulating both before and after taping. All participants ambulated across the GaitRite carpet three times and completed a visual analogue scale pain score for each trip.

Results

The results of our study found there to be no significant difference in the cadence for gait between the participants without PFPS and participants with PFPS (105.2 seconds vs. 105.1 seconds, p = 0.272), or in the stance time between the control and PFPS group (1.43 seconds vs. 1.44, p = 0.907). Similarly, no significant difference was found in participants with PFPS before and after Kinesio Taping in the cadence and stance times (105.1 seconds vs. 107.4 seconds, p =0.288, and 1.44 vs. 1.40, p = 0.272). There was a significant difference in pain in PFPS participants before and after taping, with a 112.5% reduction in pain reported after taping (3.4 vs. 1.6, p < 0.05).

Discussion and conclusion

This study is one of the first studies to utilize the GaitRite system in order to analyze the impact of Kinesio Tape on gait in participants with PFPS. While our study failed to demonstrate a significant difference in the gait of participants with PFPS in comparison to those without PFPS, we did demonstrate a significant reduction in pain after the application of Kinesio Tape. These results suggest other variables addressed by the Kinesio Tape may be causing the pain associated with PFPS.

## Introduction

One of the most common musculoskeletal conditions treated by orthopedists and physical therapists is patellofemoral pain syndrome (PFPS) [1]. Unlike chronic conditions like osteoarthritis that are due to years of mechanical stress and “trauma” culminating in the erosion of the joint surface, PFPS primarily occurs in young, active individuals, and can be difficult to diagnose, let alone treat [2]. Currently, diagnosis relies on self-reported pain around the patella that worsens with load-bearing exercises, but with a lack of quantitative diagnostic criteria, clinicians must judge intervention or treatment benefit based on subjective information [3].

While the etiology of PFPS still remains unclear, current theories suggest PFPS to be the result of a combination of overuse, musculoskeletal strength imbalances, and anatomical differences creating abnormal patella tracking and forces placed on the knee [4]. This malalignment of the patella into a faulty position on the femur impacts the proper biomechanics of the extensor mechanism and may be associated with maladaptive movement patterns [4]. Specifically, the symptoms from PFPS are due to the result of excessive patellofemoral joint stress leading to abnormal patella tracking and excessive patellofemoral joint stress leading to further abnormal patella tracking [5]. The patellofemoral joint reaction forces during the load acceptance component of the stance phase is a summation of the quadriceps force and knee flexion [6].

One commonly used and acceptable intervention for treating PFPS is patellar taping devised by McConnell [7]. McConnell taping is a specific technique that moves the patella into a more favorable biomechanical alignment, facilitating proper firing of the extensor mechanism, with the goal of decreasing anterior knee pain often being achieved immediately [8,9]. A second taping approach used in the treatment of PFPS is Kinesio taping [7]. The goals of kinesio taping are to (1) to facilitate an increase in blood and lymphatic flow, (2) correct muscle function by reconditioning abnormal muscle extension and strengthening weakened muscle, (3) neurologically suppress pain by placing tape over the affected region, and (4) reposition the “dislocated” joint bone that is creating abnormal stress on muscles and fascia [10]. While less commonly used than McConnell taping, participants treated using Kinesio taping for PFPS have demonstrated improvements in pain scores, patella alignment, and strength comparable to participants treated with McConnell taping [7].

In addition to the overt pain cause by PFPS, participants also experience changes in their gait that may further exacerbate the pain. Reduced speed of walking and reduced knee flexion are believed to be compensatory mechanisms used by individuals with PFPS to reduce the load on the patellofemoral joint [6]. PFPS participants have demonstrated abnormal gait patterns with decreased stance phase knee flexion, decreased walking velocity, less passive hip range of motion, and decreased muscle activity of the quadriceps musculature, and restoration of normal gait kinematics may be an important component of improving function [11-13]. Yet despite the increasingly more common utilization of Kinesio taping in the treatment of participants with PFPS, and potential for Kinesio Tape to improve gait, no studies have assessed PFPS participants’ gait before and after the application of Kinesio tape. Knowledge of the effects of Kinesio taping on the spatial and temporal parameters of the gait cycle in individuals with PFPS would provide additional information about the usefulness of Kinesio taping as a treatment intervention for improving gait function. Therefore, the purpose of this study was (1) to determine whether individuals with PFPS have altered spatial and temporal parameters of gait and (2) whether Kinesio taping improved their spatial and temporal parameters in their gait cycles through the use of a the GaitRite system, a temporospatial gait analysis system.

## Materials and methods

Study design

This is a prospective, controlled, single-center, non-blinded randomized trial to determine the effect of PFPS on gait and the effect of Kinesio taping on the spatial and temporal parameters of the gait cycle. This study was approved by our institution’s review board.

Participants

Criteria for inclusion into the study were: female gender, and age 18-35 years old. Criteria for exclusion from the study was the presence of knee pain in participants without PFPS, prior knee surgery to the symptomatic knee, a neurological condition that would influence ambulation, any ligamentous instability of the symptomatic knee that would influence gait and a history of traumatic patellar dislocation. Standard orthopedic tests were utilized to exclude those participants who had ligamentous pathology, including Varus-Valgus stress tests, Lachman’s test, Anterior drawer test, Posterior drawer test and Sag sign. Standard orthopedic tests were utilized to exclude those participants with meniscal pathology including McMurray’s test and Bounce Home test. Neurological screening of Quadriceps Deep Tendon reflex was utilized to exclude participants with a neurological condition that would influence ambulation. Participants for the experimental group were required to have anterior knee pain during ambulation and anterior knee pain with any of the following activities: descending stairs, prolong sitting, during jumping, with kneeling, during isometric contraction of the quadriceps musculature, and with squatting. Ten female participants participated in the study. Participants were stratified into the PFPS or control group based on their status of PFPS diagnosis.

Sample size

A convenience sample was utilized for this study, with participants recruited from local orthopaedic surgeons, and physical therapy clinics. Each participant in the control group was informed that they would be required to ambulate three times on the GaitRite carpet. Each participant in the PFPS group served as their own control and was required to ambulate three times on the GaitRite carpet with their symptomatic knee un-taped and then ambulate three times on the GaitRite carpet with their symptomatic knee taped with Kinesio Tape.

Intervention

Participants performed the first set of ambulation trials without tape to capture their natural compensation during the gait cycle without the influence of any intervention. All participants completed several practice trials to accommodate to the GaitRite carpet and minimize the likelihood of an order effect. Participants in the control group were instructed to walk at a self-pace leading with their dominant lower extremity. The dominant leg was determined to be the leg they would kick a ball with. Participants in the experimental group were instructed to walk at a self-pace leading with the symptomatic lower extremity. One trial was defined as one complete episode of ambulation down the carpet. The control group performed three trials of ambulation on the GaitRite. The experimental group performed three trials of ambulation un-taped and three trials of ambulation taped on the GaitRite.

**Figure 1:**
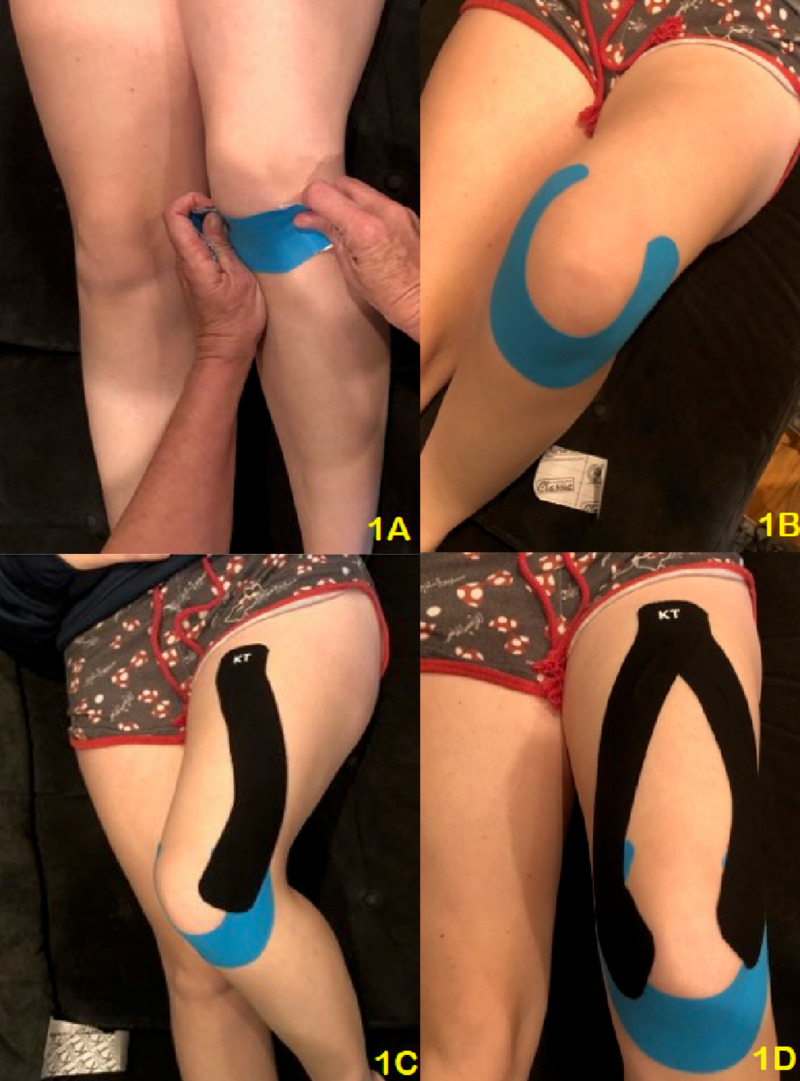
Kinesio taping of the knee utilizing a "U" and "Y" technique (1A) Initiate taping using the "U" technique at the inferior pole of the patella. (1B) Finish "U" with ends of the Kinesio Tape 2-3 cm superior to the femoral condyles. (1C) Begin the "Y" technique at the proximal aspect of the thigh, and extend to the lateral aspect of the knee. (1D) Applying a second piece, complete the "Y" finishing at the medial aspect of the knee.

To ensure standardization of the taping technique, the principal investigator completed Kinesio taping continuing education offered by the Kinesio Taping Association. The principal investigator practiced the taping techniques to establish the required uniformity and appropriate tension demanded. The principal investigator has over 17 years of clinical experience in the evaluation and treatment of patellofemoral pain at the time of this study. The Kinesiotaping Association established guidelines for preparing the area for taping were followed [14]. A combination of two taping techniques was used for mechanical correction. The first technique utilized the “U-technique” for mechanical correction and was applied around the patella to facilitate a tilting effect, decreasing pressure on the inferior pole of the patella (Figure 1). Application of this taping technique first, required the Kinesio Tape to be applied with 50% tension over the patella inferior pole. Next, the participant flexed the knee with the medial end of the tape, with 15% tension in the tape, was applied over the vastus medius and the lateral end of the tape, with 25% tension, was applied over the vastus lateralis. The elastic qualities of the tape with the “Y-technique” for mechanical correction for muscle weakness or to facilitate more contraction on the quadriceps musculature. The tape is applied around the muscle starting at the origin and finishing at the insertion with 25% tension in the base and in the tails of the tape.

Participants performed the first set of ambulation trials without tape to capture their natural compensation during the gait cycle without the influence of any intervention. All participants completed several practice trials to accommodate to the GaitRite carpet and minimize the likelihood of an order effect. Participants in the control group were instructed to walk at a self-paced gait leading with their dominant lower extremity. The dominant leg was determined to be the leg they would kick a ball with. Participants in the experimental group were instructed to walk at a self-paced gait leading with the symptomatic lower extremity. One trial was defined as one complete episode of ambulation down the carpet. The control group performed three trials of ambulation on the GaitRite. The experimental group performed three trials of ambulation un-taped and three trials of ambulation taped on the GaitRite.

Outcomes

For each participant, measurements of bilateral leg length (distance from the center of the anterior superior iliac spine to the inferior aspect of the medial malleolus) were taken. Additionally, a visual analogue scale (VAS) to determine the level of pain with a scale of 0-10 in participants with PFPS post-ambulation with an untaped knee and then again post-ambulation with a taped knee taped. The VAS was chosen as it has been used in previous PFPS studies assessing the impact of Kinesio Tape on pain [15]. The temporal and spatial parameters of gait for this study were measured utilizing the GaitRite system (CIR Systems Inc., Clifton, NJ). The GaitRite system is a portable electronic walkway. The carpeted walkway has an active area that is 61 cm wide and 366 cm long. Within this active area are 13,824 pressure sensors arranged in a horizontal grid. As the participant walks across the carpet, the pressure sensor close enabling the collection of the spatial and temporal gait parameters. Through a series of cables, the walkway is connected to an IBM compatible computer. The spatial and temporal parameters of gait were automatically calculated using the GaitRite software.

Statistical analysis

The cadence, stance time, VAS pain score pre- and post-taping were recorded for each participant. A paired-test and independent t-test were used to determine if a statistically significant difference existed between the outcome measures, utilizing a p-value of <0.05 to indicate significance. Statistical analysis was performed using the Statistical Package for the Social Sciences (SPSS) (IBM, Armonk, NY).

## Results

Totally, 10 participants were recruited for this study; five in the control group and five participants were in the PFPS group. Participant’s ages ranged from 20 to 35 years with the mean age of 29.6 ± SD of 4.1, and all participants were Caucasian females.

The results showed no significant difference in the cadence for gait between the participants without PFPS and participants with PFPS. The average cadence in participants without PFPS was 105.2 seconds, and in participants with PFPS 105.1 seconds (p = 0.272) (Table 1). Furthermore, no significant difference in cadence was found between the gait cadence of participants with PFPS before Kinesio taping and their cadence after Kinesio Taping, 105.1 seconds vs. 107.4 seconds, p = 0.288 (Table 2).

**Table 1:** Cadence and stance time in control versus patellofemoral pain syndrome subjects

	Mean Cadence Time (Seconds)	P-Value	Mean Stance Time (Seconds)	P-Value
Control (SD)	105.2 (8.09)	p = 0.272	1.43 (0.117)	p = 0.288
Subjects with Patellofemoral Pain Syndrome (SD)	105.1 (12.6)		1.44 (0.169)	

**Table 2:** Cadence, stance time, and pain in patients with patellofemoral pain syndrome after ambulating before and after Kinesio taping

	Before Kinesio Taping (seconds)	After Kinesio Taping (seconds)	P-Value
Mean Cadence (SD)	105.1 (12.6)	107.4 (10.6)	0.288
Mean Stance Time (SD)	1.44 (0.169)	1.40 (0.149)	0.272
Visual Analogue Scale Pain Score (SD)	3.4 (1.67)	1.60 (1.51)	0.037

There was no significant difference between the time spent in the stance phase in participants without PFPS and participants with PFPS (1.43 seconds vs. 1.44, p = 0.907). Furthermore, no significant difference was found in the stance time of participants with PFPS before and after Kinesio taping, 1.44 vs. 1.40, p = 0.272.

A statistically significant difference in pain scores was found in participants with PFPS before and after Kinesio taping, accounting for a more than 112.5% reduction in pain score (3.4 vs. 1.6, p < 0.05). VAS pain was not recorded for participants in the control group as knee pain was a criterion for exclusion.

## Discussion

While a multitude of studies have analyzed the efficacy and benefit of Kinesio Taping in participants with PFPS, no studies have analyzed the spatial and temporal parameters of gait using gait analysis software pre- and post-taping. Our study is one of the first studies to specifically analyze the cadence and stance phases of gait in participants without PFPS and participants with PFPS and assess the impact of Kinesio Tape on gait in PFPS participants.

The results of our study failed to find a significant change in the cadence and stance time participants with PFPS. Although no direct comparison can be made due to different patella taping techniques utilized in clinical practice, the reduction in pain with no significant change in gait cadence in this study does not correlate with other authors findings [5,11,12]. The finding of the study conducted by Salsich et al. using McConnell patellar taping with PFPS participants found a direct correlation with a decrease in pain and an increase in cadence, as well as an increase in knee extensor moments during stair ambulation [11]. This may be due to the multifactorial etiology of PFPS, with no clear etiology being currently established in the literature, and the presence of multiple possible mechanisms for PFPS, some of which do and do not impact cadence and stance time [2,4,12]. As such, the effects of Kinesiotaping on quadriceps musculature and the extensor mechanism remains unclear. In this study, the quadriceps musculature did not respond as anticipated with no changes noted in the cadence or stance time during gait. Studies have found quadricep musculature weakness in participants with PFPS and may be a confounding variable when assessing the efficacy of Kinesio taping on gait in participants with PFPS [5].

The results of this study indicate that Kinesio taping produced a significant effect on pain reduction in participants with PFPS compared to untaped when tested for stance time and cadence using the GaitRite. The results of this study of Kinesio taping resulting in the reduction of pain after the application support the well-documented reduction in pain after the application of Kinesio tape to multiple areas of the body, including the knee [16,17]. Although no direct comparison can be made, the results of this study are also similar to the results of other studies conducted for pain reduction of PFPS using McConnell taping. As mentioned earlier, several investigators have found a reduction in pain after patella taping. A possibility of why a reduction in pain occurred in these participants with no change in stride time or cadence could be the result of this particular application technique of the Kinesio tape. The mechanical correction technique did not change the space position of the patella and therefore did not facilitate an improvement in the knee flexion angle, which would alter the quadriceps dynamics in the gait cycle. The application of the mechanical correction technique with the Kinesio tape possibly lead to a different muscle strategy utilized during gait and therefore a reduction in pain was noted but no change in cadence or stride time.

While we believe that our study could have a positive impact on the clinical decision making with respect to the treatment and management of participants with PFPS, we acknowledge it is not without limitations. First and foremost, the generalizability and results of our study are limited by our sample size. Furthermore, while PFPS is more common in females than in males, the inclusion of only female participants may further limit the generalizability of the study. Additionally, the incorporation of musculoskeletal strength testing to assess for quadriceps strength may have allowed for the identification of potential confounding factors.

Despite the limitations, our study has several strengths. First and foremost, this is one of the first studies to use the GaitRite video analysis system to analyze the gait patterns of participants with PFPS before and after Kinesio taping. Additionally, our study further bolsters the literature supporting the use of Kinesio taping in the treatment of PFPS and lays the groundwork for future studies utilizing the GaitRite system to assess Kinesio taping and PFPS. Further studies could include expanding the study of the spatial and temporal parameters of gait using Kinesio taping.

## Conclusions

This study demonstrated that participants with PFPS during ambulation with applied Kinesio taping produced a significant reduction in reported pain. The effect measured for pain reduction was for an immediate relief only. Despite Kinesio taping resulting is a significant reduction in PFPS pain, no significant findings were noted for changes in cadence or stance time in participants with PFPS suggesting that deficits in quadriceps recruitment still may have been present. This study suggests that Kinesio taping with a mechanical technique is effective for pain relief that may not be the result of alterations in a participant’s gait.
